# Letter to Editor on “L-shaped association of selenium in blood metal mixtures with the incidence of liver fibrosis/cirrhosis: NHANES 2017–2020”

**DOI:** 10.1097/JS9.0000000000004769

**Published:** 2026-01-21

**Authors:** Yamei Wu

**Affiliations:** Reproductive Medicine Center, Hainan Women and Children’s Medical Center, Haikou, China

*To the Editor,*

Wang *et al* examine an important and underexplored question – how metal-mixture exposures relate to liver fibrosis/cirrhosis at the population level – leveraging NHANES 2017–2020 with vibration-controlled transient elastography (VCTE)^[1]^. By applying multiple mixture frameworks (WQS, quantile g-computation, BKMR) alongside spline-based dose–response modeling, the authors consistently identify selenium as the dominant component inversely associated with fibrosis/cirrhosis, with an “L-shaped” relationship and an apparent benefit plateau^[1]^. We prepared this commentary in accordance with the TITAN 2025 guideline to ensure transparent and responsible reporting^[2]^.

These results are biologically and clinically plausible. Selenium contributes to antioxidant defense and redox homeostasis, and prior NHANES-based evidence suggests higher serum selenium is associated with a lower prevalence of advanced fibrosis and lower all-cause mortality in NAFLD – supporting a protective signal, at least within typical exposure ranges^[3]^. The current study extends this concept by explicitly modeling mixtures, which better reflect real-world exposomes than single-metal analyses^[1]^.

From a surgical standpoint, the work is thought-provoking because fibrosis/cirrhosis materially increases perioperative risk and influences procedural selection, bleeding risk, infection, and decompensation. Contemporary data also support VCTE-derived scores as clinically meaningful predictors of liver-related events, strengthening the relevance of elastography-based phenotyping in large-scale studies^[4]^. In NAFLD/MASLD-prevalent populations – where subclinical fibrosis is common and guideline-endorsed risk assessment is increasingly emphasized – an inexpensive biomarker such as blood selenium could, if validated, complement existing preoperative risk stratification pathways^[5]^.

Several limitations should temper immediate translation. The analysis is cross-sectional, so reverse causation remains plausible (chronic liver disease may alter trace element handling). VCTE thresholds can misclassify fibrosis stages, and the study focuses on a restricted set of metals, leaving broader dietary, environmental, and occupational exposures incompletely captured^[1]^. The mediation effects are modest and should not be interpreted as mechanistic proof. Importantly, the “L-shape” also cautions against simplistic supplementation messaging; excessive selenium intake is a recognized cause of toxicity, as highlighted by a well-documented outbreak linked to misformulated supplements^[6]^.

Overall, Wang *et al* provide a compelling population-level signal that selenium within blood metal mixtures is inversely associated with elastography-defined fibrosis/cirrhosis^[1]^. Prospective cohorts with repeated measures, detailed dietary/supplement data, broader metal panels, and perioperative outcomes are now needed to clarify causality, define safe target ranges, and determine whether selenium meaningfully improves clinical risk prediction.

## Data Availability

No data was used in this Letter to the Editor.
